# Neuroinflammation, Pain and Depression: An Overview of the Main Findings

**DOI:** 10.3389/fpsyg.2020.01825

**Published:** 2020-07-31

**Authors:** Ana Carolina Pinheiro Campos, Geiza Fernanda Antunes, Marcio Matsumoto, Rosana Lima Pagano, Raquel Chacon Ruiz Martinez

**Affiliations:** ^1^Division of Neuroscience, Hospital Sirio-Libanes, São Paulo, Brazil; ^2^Anesthesiology Medical Center, Hospital Sirio-Libanes, São Paulo, Brazil; ^3^LIM 23, Institute of Psychiatry, University of São Paulo School of Medicine, São Paulo, Brazil

**Keywords:** neuroinflammation, pain, depression, depression-pain syndrome, glial cells

## Abstract

Chronic pain is a serious public health problem with a strong affective-motivational component that makes it difficult to treat. Most patients with chronic pain suffer from severe depression; hence, both conditions coexist and exacerbate one another. Brain inflammatory mediators are critical for maintaining depression-pain syndrome and could be substrates for it. The goal of our paper was to review clinical and preclinical findings to identify the neuroinflammatory profile associated with the cooccurrence of pain and depression. In addition, we aimed to explore the regulatory effect of neuronal reorganization on the inflammatory response in pain and depression. We conducted a quantitative review supplemented by manual screening. Our results revealed inflammatory signatures in different preclinical models and clinical articles regarding depression-pain syndrome. We also identified that improvements in depressive symptoms and amelioration of pain can be modulated through direct targeting of inflammatory mediators, such as cytokines and molecular inhibitors of the inflammatory cascade. Additionally, therapeutic targets that improve and regulate the synaptic environment and its neurotransmitters may act as anti-inflammatory compounds, reducing local damage-associated molecular patterns and inhibiting the activation of immune and glial cells. Taken together, our data will help to better elucidate the neuroinflammatory profile in pain and depression and may help to identify pharmacological targets for effective management of depression-pain syndrome.

## Introduction

Chronic pain is a complex disorder that significantly impacts society and is the leading cause of disability and financial burden worldwide (Global Burden of Disease Study 2016). It is considered a public health problem and affects approximately 20% of the general population (Breivik et al., 2006). This type of pain is defined as that is persistent or intermittent pain that lasts for more than 3 months despite a normal tissue healing time (Merskey and Bogduk, 1994). Nociceptive pain may occur as a consequence of non-neural damage (Cohen and Mao, 2014), while neuropathic pain is induced by lesions or diseases involving the central nervous system (CNS) (Li et al., 2016). The prevalence of chronic pain ranges from 7 to 10% of the general population (van Hecke et al., 2014), and this disorder manifests as spontaneous pain, hyperalgesia and mechanical allodynia, which decrease quality of life (Baron, 2000). Because of its association with poor quality of life, chronic pain often induces depression (von Knorring et al., 1983; Lee et al., 2009; Agüera-Ortiz et al., 2011). Depression can be characterized by psychological and physical symptoms, including low mood or sadness, lack of energy, lack of motivation, insomnia, low sex drive and an inability to enjoy life (Cui, 2015). Depression is also considered a public health problem and is one the major contributors to global disease burden (Collins et al., 2011; Whiteford et al., 2013). Chronic pain may induce depression, and people suffering from depression may also present abnormal pain perception and modulation, which increases the risk of developing chronic pain (Currie and Wang, 2005). It has been estimated that 85% of people affected with chronic pain suffer from severe depression (Bair et al., 2003; Williams et al., 2003), supporting the concept that both conditions coexist and exacerbate one another (Gallagher and Verma, 1999; Blier and Abbott, 2001; Leo, 2005). This association has been labeled depression-pain syndrome or the depression-pain dyad (Lindsay and Wyckoff, 1981; Beckman, 2004; Cox et al., 2017).

A possible reason why these disorders are complex and difficult to treat is that they both involve neuroinflammation. Neuroinflammation is an innate immune response of the nervous system to injury, infection or neurodegenerative disease characterized by the activation of resident glial cells, including microglia and astrocytes; release of cytokines and chemokines; and activation and infiltration of leukocytes (Takeuchi and Akira, 2010; Domercq et al., 2013; Ji et al., 2014, 2016, 2018; Turner et al., 2014; Stephenson et al., 2018; Yang and Zhou, 2019). It has also been proposed that these conditions could be responsible for alterations in the permeability of the blood-brain barrier, immune cell infiltration, and activated microglia (Benatti et al., 2016). Persistent neuroinflammation plays an important role in the induction and maintenance of depression-pain syndrome (Ji et al., 2014; Burke et al., 2015). In this sense, classically activated resident glial cells lose the ability to control the synaptic environment (Liddelow et al., 2017). Specifically, improvements in the synaptic environment could promote symptomatic progress and modify disease progression (Pecina and Zubieta, 2018). However, the relationship between inflammation and the synaptic environment in depression-pain syndrome has been poorly investigated (Liddelow et al., 2017).

In the literature, much effort has been devoted to fully understanding the pathophysiology of chronic pain and depression as well as understanding why comorbidity of these two disorders is so common to guide the development of better treatments. It is not surprising that pain relief medications are still the second-most prescribed drugs (after cardiac-renal drugs) in the United States (Turk, 2002; Van Zee, 2009; Dowell et al., 2016; Volkow and McLellan, 2016). Pharmacological treatment options for chronic pain include opioids, non-steroidal anti-inflammatory drugs, gabapentinoids, tricyclic antidepressants, serotonin-norepinephrine reuptake inhibitors, NMDA antagonists, and topical preparations (Turk, 2002; Cooper et al., 2017; Eccleston et al., 2017; Manchikanti et al., 2017; Riediger et al., 2017; Shanthanna et al., 2017; Szok et al., 2019). The use of opioids and non-steroidal anti-inflammatory drugs is limited by their adverse effects, tolerance, and potential for addiction (Barber and Gibson, 2009; Han et al., 2015; Vowles et al., 2015; Busse et al., 2018). Additionally, considering that chronic pain treatment frequently involves polypharmacy therapy, it is associated with an increased risk of drug interactions and additive adverse effects (Barber and Gibson, 2009; Taylor et al., 2013; Denk et al., 2014; Riediger et al., 2017; Busse et al., 2018). Furthermore, despite the range of drugs available, pharmacological therapies for chronic pain are often inefficient for the management of depression-pain syndrome, and as this condition can affect nearly every aspect of daily life; thus, the development of novel treatment strategies is critical (Bentley et al., 2014; Cohen and Mao, 2014; Crofford, 2015; Brant et al., 2017; Shamji et al., 2017). Treatment focusing on neuroinflammatory substances could help to inhibit glial cells in their activated state, improve the neuronal network in specific regions and induce alleviation of chronic pain and depression (Walker et al., 2013a; Ji et al., 2014, 2018). On the other hand, it has been hypothesized that treatments that modulate the neuronal network may decrease the formation of glial cell-recognized patterns and decrease inflammation, consequently modulating the symptoms of depression-pain syndrome. In this sense, the goal of our paper was to review clinical and preclinical findings to identify the inflammatory signature associated with depression-pain syndrome and the different types of treatments that modulate neuroinflammation. We hope to discuss the anti-inflammatory mechanisms observed in the studied articles and improvements in the neurocircuitry, which may be responsible for the optimal modulation of these symptoms.

## Methods

### Search Strategy and Inclusion and Exclusion Criteria

A quantitative review search of PubMed, ScienceDirect and MEDLINE for original research articles supplemented by manual screening was conducted between November and December 2019. As this study aimed to review many published articles on the topics of neuroinflammation and pain-depression syndrome, there were no restrictions placed on the publication date for the search. Thus, we opted to not conduct a formal systematic review or meta-analysis. The studies were required to contain the relevant terms “neuroinflammation AND chronic pain AND depression OR glial cells AND chronic pain AND depression OR chronic pain AND depression OR negative effects AND patients AND animal model OR chronic pain AND depression AND animal models AND patients.” The selection criteria included studies with (1) clinical and preclinical data; (2) human and rodent data; and (3) cooccurrence of pain, depression and neuroinflammation. Studies of all sample sizes were included in the analysis. Studies were excluded if they (1) were reviews or meta-analyses; (2) presented repeated data from previously included studies or (3) were not published in English.

**FIGURE 1:**
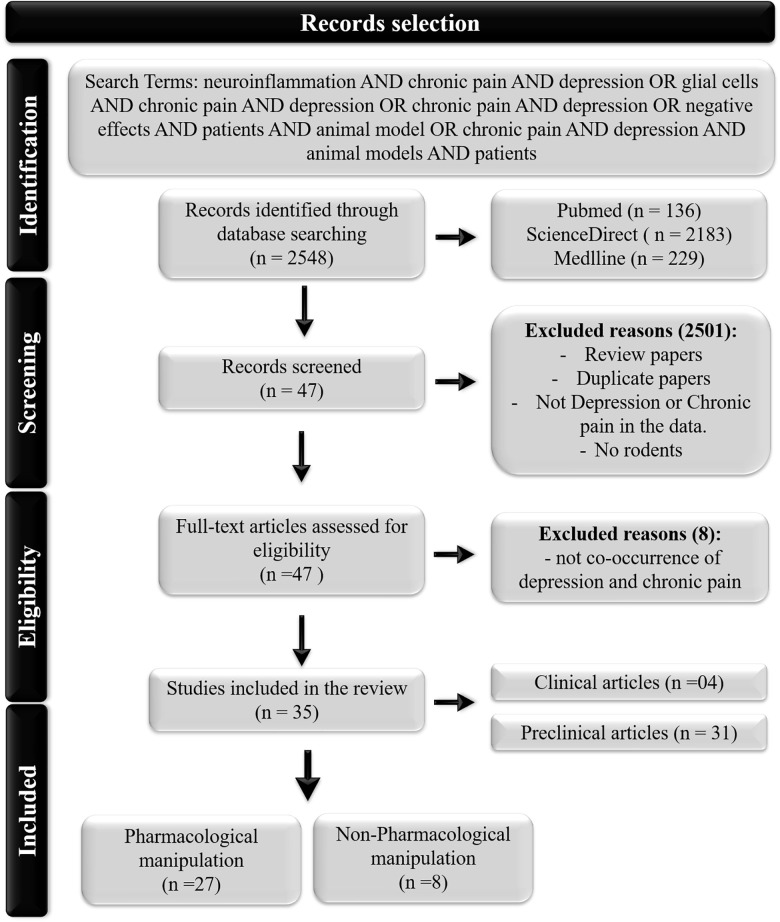
Flowchart of the study.

As shown in Figure 1, the search yielded 2,548 records. We eliminated articles that did not investigate the cooccurrence of painful and depressive behaviors or did not investigate any inflammatory biomarkers. Ultimately, 35 studies were included in this review.

## Results

### Target Structures Involved in Pain and Depression

From the 35 selected papers, we found 31 and 4 reports of preclinical models and clinical investigations, respectively. Eight of the selected articles evaluated the inflammatory signature in the presence of painful and depressive behavior, while 27 articles evaluated the inflammatory response related to different types of treatments. The structures that play an important role in pain and depression states listed in the selected papers included the cerebral cortex and its subdivisions, the prefrontal cortex (PFC), the anterior cingulate cortex (ACC), the amygdala, the hippocampus, and the raphe nuclei, as alterations in plasma were observed in human subjects.

The main general markers of the cooccurrence of chronic pain and depression were related to central inflammation, particularly, tumor necrosis factor (TNF)-α, interleukin (IL)-1β, and IL-6, as well as peripheral discrepancies in cortisol levels. Regarding treatment for depression-pain syndrome, we found that therapies with different mechanisms may attenuate central and peripheral inflammation, raising a discussion about the relationship between the neuronal network and inflammation modulation.

## Discussion

### Target Structures Involved in Pain and Depression

During pain processing, structures such as the PFC, ACC, nucleus accumbens (NAc), thalamus, hippocampus and amygdala send projections to the periaqueductal gray (PAG) to activate the descending analgesic pathway, including the raphe nucleus and locus ceruleus (Fields and Basbaum, 1999; Apkarian et al., 2005; Seminowicz et al., 2019). These areas are connected and often modulate one another, as first described by Papez (1937). The original Papez circuit consists of the hippocampus, thalamus, cingulate gyrus and cerebral cortex; however, further structures have been added over the years, such as the insular cortex, amygdala, and other subcortical nuclei (Yakovlev, 1948; Nakano, 1998). In this sense, the limbic system, a network of brain structures involved in emotion, memory, learning and homeostatic processes that strongly affect behavior (Arora et al., 2011; Catani et al., 2013), suggesting a major correlation between pain and depression.

Therefore, many authors have searched for inflammatory signatures in limbic system structures, especially the PFC and hippocampus. It has been proposed that the expression of genes encoding proinflammatory cytokines are increased in these limbic structures responsible for pain processing and depression (Arora et al., 2011; Kim et al., 2012). A structure mentioned in 24% of the articles was the PFC. The medial PFC (mPFC), which is composed of the granular and agranular areas, including the ACC (Ong et al., 2019), was also investigated in the articles we found. Additionally, another 12% of the articles described the signature in the cerebral cortex without specifying which regions were involved. Some authors probably preferred to address the structures more generally due to the methodological difficulty of ensuring the extraction if the specific area of the brain from fresh tissue. The mPFC sends projections to the PAG (Bragin et al., 1984; An et al., 1998) that project to the nuclei raphe and are involved in the analgesic descending pathway (Fields and Basbaum, 1999; Apkarian et al., 2005; Seminowicz et al., 2019). The mPFC is important for pain processing and mediates antinociceptive effects due to its connections with other cortical areas and the PAG (Ong et al., 2019). However, increased and persistent activation of the thalamus, which projects to the somatosensory cortex and the PFC, mediates the chronification of pain, possibly via corticostriatal projections, dopaminergic system dysfunction and ventral tegmental area (VTA)-NAc reward pathways (Ong et al., 2019). Additionally, although the PFC is often thought of as the center of thinking and decision making, it is also associated with depression (Treadway et al., 2015). Lesions of the mPFC, which is responsible for affection and negative emotion (Miller and Cohen, 2001), may attenuate depression by decreasing the direct activation of the amygdala (Ellenbogen et al., 2005; Sachdev and Sachdev, 2005; Connolly et al., 2017).

It has been proposed that environmental stress can contribute to the development of depression through inflammatory and epigenetic mechanisms (Slavich and Irwin, 2014; Park et al., 2019). Specifically, the PFC and amygdala have opposite responses to stress since synaptic plasticity is enhanced in the amygdala after depression and is decreased in the PFC (Marsden, 2013). Inhibition of the amygdala by local GABAergic agonists changes pain sensitivity and depressive-like behavior (Seno et al., 2018). Additionally, the volume of the amygdala has been suggested to be correlated with the severity of depression (Li et al., 2014), demonstrating the importance of its activation in the pathophysiology of depression.

Persistent pain may cause dystrophy of hippocampal areas by reducing volume, increasing abnormal cytokine expression, and inducing deficits in long-term potentiation as well as impairing neurogenesis (Duric and McCarson, 2005; Kodama et al., 2007; Terada et al., 2008; Al-Amin et al., 2011; del Rey et al., 2011; Erickson et al., 2011; Mutso et al., 2012). These alterations can induce anxiety and stress since the hippocampus and PFC play critical roles in regulating the hypothalamus-pituitary-adrenal (HPA) axis (Jacobson and Sapolsky, 1991). After HPA activation, glucocorticoid hormones (corticosterone in rodents and cortisol in humans) are released from the adrenal gland in response to stress and easily penetrate into the human brain (Karssen et al., 2001). The hippocampus, PFC and brainstem monoaminergic nuclei strongly respond to the increased levels of these glucocorticoids (Reul and de Kloet, 1985). In this sense, dopamine release is increased in acute stress (Castro and Zigmond, 2001), while it is downregulated after chronic exposure, causing modulation of the VTA-NAc reward pathway (Thompson et al., 2004). Additionally, chronic exposure to corticosterone/cortisol induces hippocampal dendritic atrophy (Landfield et al., 1981; Meaney et al., 1988), suggesting that depression and anxiety can induce changes in neural plasticity in areas also involved in controlling the nociceptive system and may “predispose” the brain to persistent pain sensitivity. The majority of the articles we found investigated the hippocampus, demonstrating the importance of this region in pain, depression, and depression-pain-syndrome. We also found that some authors investigated the dorsal root of the ganglia (DRG) and the spinal cord, structures strongly related to pain control (Todd, 2010; Krames, 2014), especially in models of pain induced by nerve damage.

**FIGURE 2:**
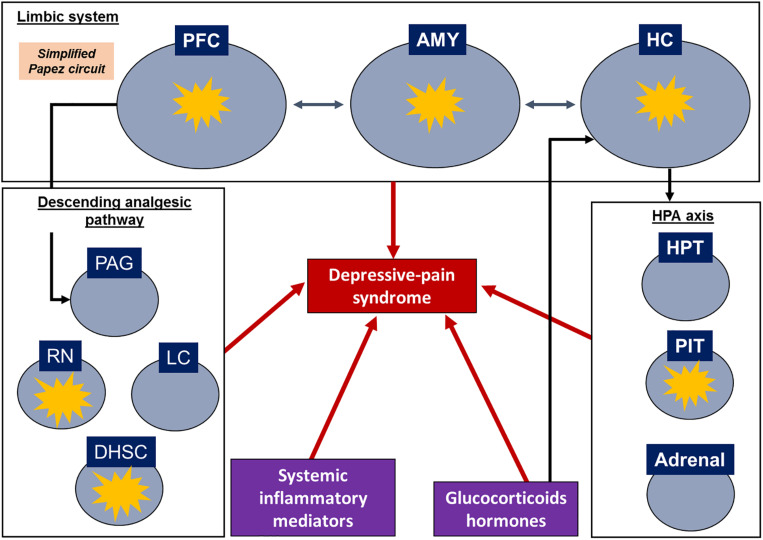
Schematic representation of depressive-pain syndrome and the relationship between target structures and mediators. Target structures with inflammatory signatures in the identified articles. AMY, amygdala; DHSC, dorsal horn of the spinal cord; HC, hippocampus; HPT, hypothalamus; LC, locus coeruleus; PAG, periaqueductal gray matter; PFC, prefrontal cortex; PIT, pituitary; RN, raphe nuclei.

Regarding the assessment of systemic samples, some articles evaluated corticosterone levels and some inflammatory biomarkers in the serum or plasma. Plasma and serum are more related to the neuroendocrine response and peripheral inflammation since the blood-brain barrier (BBB) can prevent the ability of substances in the blood circulation to access the CNS (Daneman and Prat, 2015); however, some studies have noted that neuroinflammatory settings may compromise the integrity of the blood-nerve barrier and alter the BBB (Skaper, 2016; Schaefer et al., 2017). A schematic review of target structures and inflammatory signatures is shown in Figure 2.

### Neuroinflammatory Signature of Preclinical Models (Table 1)

In the peripheral nervous system, classic inflammatory modulators are secreted by neutrophils in a more acute stage of neuroinflammation and then by macrophages and T cells after the persistence of inflammation sensitizes either the nociceptor sensory receptors (Basbaum et al., 2009) or peripheral glial cells, such as Schwann cells and satellite glia cells (Ellis and Bennett, 2013), to potentially noxious stimuli in primary afferent neurons, modulating pain sensitivity (Ji R. R. et al., 2013). Infiltrating macrophages and Schwann cells can secrete proinflammatory cytokines, such as IL-1β and TNF-α (Nathan, 1987; Palmer and Weaver, 2010), which is consistent with the increase in plasma proinflammatory cytokines in people suffering from chronic pain (Vendrusculo-Fangel et al., 2019) and preclinical pain models (Table 1). Both peripheral and central inflammation are associated with symptoms of pain and depression (Walker et al., 2013b), as characterized by dysregulation of the immune system (Burke et al., 2014), neurotransmitters such as noradrenaline and serotonin (Stahl and Briley, 2004; Goldenberg et al., 2010), neuropeptides such as brain-derived neurotrophic factor (BDNF) and glial cell line-derived neurotrophic factor (GDNF) (Kramer et al., 1998; Werner and Coveñas, 2010), oxidative stress (Arora et al., 2011) and cytokines (Wallace, 2006; Dowlati et al., 2010).

**TABLE 1:** Inflammatory signature upon presence of painful and depressive behavior in preclinical and clinical research.

*Pain models investigating depression*					
References	Pain model	Painful behavior	Depressive-like behavior	Inflammatory effects
González-Sepúlveda et al., 2016	PSNL	↓ Thermal nociceptive threshold using the cold plate test	↓ Time spent in open arms in the EPM	↑ IL-1 β in the PFC
			↑ Murbles buried	
		↓ Mechanical nociceptive threshold using the von Frey test	↑ Immobility in the tail suspension	
			↑ Alcohol drinking in the dark	

Gonçalves et al., 2008	SNI	↓ Mechanical nociceptive threshold using the von Frey and pin-prick tests	↑ Immobility in the FST	↑ BrdU, BrdU + GFAP and BrdU + calbidin labeling in the amygdala
				No change in the BrdU + NeuN labeling in the amygdala

***Depressive-live models investigating pain***					
**References**	**Depressive-like model**	**Painful behavior**	**Depressive-like behavior**	**Inflammatory effects**

Zhao et al., 2019	CUMS	↓ Thermal nociceptive threshold using the Hargreaves test	↓ Sucrose preference	↑ IL-1β and caspase-1 in the PFC and pituitary
				↑ IL-1β, IL-6 and caspase-1 in the hippocampus
		↓ Mechanical nociceptive threshold using the von Frey test	↑ Immobility in the FST	↑ TNF-α, IL-1β, IL-6 and caspase-1 in the raphe nuclei;
				↑ TNF-α, IL-1β, IL6 and CASP1 in serum
				↑ACTH and corticosterone in serum
	
	CORT	↓ Thermal nociceptive threshold using the Hargreaves test	↓ Sucrose preference	No change in the PFC
				↑ TNF-α, IL-1β, IL-6 and caspase-1 in HC
				↑ TNF-α in the raphe nuclei;
		↓ Mechanical nociceptive threshold using the von Frey test	↑ Immobility in the FST	↑ TNF-α, IL-1β, IL-6 and caspase-1 in the pituitary
				↑ TNF-α, IL-1β, IL6 and CASP1 in serum
				↑ACTH and corticosterone in serum

Mizoguchi et al., 2019	Maternal separation	↓ Thermal nociceptive threshold using the acetone test	↑ Immobility time in the FST	↑ Iba-1 (microglia activation) in the DHSC
			↓ Sucrose preference	
		↓ Mechanical nociceptive threshold using the von Frey test	↑ Performance in social interaction	
			↓ Time spent in open arms in the EPM	
López-Granero et al., 2017	Two BXD RI mouse strains, BXD21/TyJ RI, BXD84/RwwJ RI and C57BL/6 wild-type mice	Not applicable	↓ Mechanical nociceptive threshold using the von Frey test	↓ Immobility in the FST	↑TNF-α and IL-6 in serum

Dellarole et al., 2014	Wild-type	CCI	↓ Thermal nociceptive threshold using the Hargreaves test	↓ Sucrose preference	↑ TNF-α the hippocampus
			↓ Mechanical nociceptive threshold using the von Frey test	↓ Body weight	↑ TNFR2 the hippocampus
				↓ Physical state	No change in TNFR1 the hippocampus
			
	TNFR1^–/–^			↓ Sucrose preference	
			No change in thermal nociceptive threshold using the Hargreaves test	↓ Body weight	↑ TNF-α in the hippocampus
			No change in mechanical nociceptive threshold using the von Frey test	↓ Physical state	No change in TNFR2 in the hippocampus

***Pain and Depressive-live models***					

**References**	**Depressive-like model**	**Pain Models**	**Depressive-like behavior**	**Painful behavior**	**Inflammatory effects**

Burke et al., 2013	Sham	SNL	Not measure	↓ Thermal nociceptive threshold using the acetone test, but no change in Hargreaves test	No change in CD11b, GFAP, IL-1b and TNF-α in the amygdala
				↓ Mechanical nociceptive threshold using the von Frey test	
	
	OB	Sham	Not measure	↓ Thermal nociceptive threshold using the acetone test, but no change in Hargreaves test	↑ CD11b, GFAP and IL-1b in the amygdala
				↓ Mechanical nociceptive threshold using the von Frey test	
	
	OB	SNL	Not measure	↓ Thermal nociceptive threshold using the acetone test, but no change in Hargreaves test	No change CD11b, GFAP, IL-1β and TNF-α in the amygdala
				↓ Mechanical nociceptive threshold using the von Frey test	

**Clinical data**					

**References**	**Diagnosis**		**Painful behavior**	**Depressive-like behavior**	**Inflammatory effects**

Euteneuer et al., 2011	Major depression		↑ Pain sensibility using the paw pressure test	↑ Scores in BDI-II and SCL-90T GSI	No change in TNF-α and IL-6 expression in serum

*ACTH, adrenocorticotropic hormone; BDI-II, Beck Depression Inventory-II; BrdU, bromo-deoxyuridine; BXD RI, BXD recombinant inbred (RI) mice strains; BXD21/TyJ, BXD RI mouse strain; CCI, chronic constriction injury; CD11b, cluster of differentiation 11b; CORT, chronic corticosterone treatment; CUMS, chronic unpredictable mild stress; DHSC, dorsal horn of the spinal cord; EPM, elevated plus maze; FST, forced swimming test; GFAP, glial fibrillary acidic protein; HC, hippocampus; Iba-1, ionized calcium-binding adapter molecule 1; IL, interleukin; NeuN, neuronal nuclei; OB, olfactory bulbectomy; PFC, prefrontal cortex; PSNL, partial spinal nerve ligation; SCL-90T, Symptom Checklist 90 Revised; GSI, Global Severity Index; SNI, spared nerve injury; SNL, spinal nerve ligation; TNF-α, tumor necrosis factor alfa; TNFR1, tumor necrosis factor receptor 1; TNFR2, tumor necrosis factor receptor 2.*

Where and how neuroinflammation starts in the absence of a pathogen or classic injury may be a complex question. In this sense, direct injury of peripheral nerves, as investigated in 57% of the articles regarding neuroinflammation in preclinical models without treatment (Table 1), begins with peripheral inflammation due to Wallerian degeneration that occurs in the distal axonal segment following nerve injury and is characterized by a process of demyelination and disintegration, release of proinflammatory cytokines and macrophage recruitment to the nerve (Toews et al., 1998; Shamash et al., 2002; Zuo et al., 2003; Perrin et al., 2005). In peripheral nerve injury, myelinating Schwann cells start proliferating and produce proinflammatory cytokines, such as TNF-α, IL-1β and monocyte chemoattractant protein 1 (MCP-1), which together with substance P (SP) and calcitonin gene-related peptide (CGRP) act as damage-associated molecular patterns (DAMPs) that activate innate immune resident cells (Zhang and An, 2007). These patterns activate different receptors present within the membrane of resident cells to induce the release of more proinflammatory cytokines and chemokines (Takeuchi and Akira, 2010).

Increased serum levels of TNF-α and IL-6 have been shown in two different BXD mouse lineages that are commonly used as genetic resources to study neuropharmacological phenomenon (Philip et al., 2010). In BXD21/TyJ RI mice, nuclear factor erythroid 2 (Nrf2) expression is upregulated, while BXD84/RwwJ RI mice exhibit low Nrf2 expression (López-Granero et al., 2017). Considering the role of Nrf2 in activating antioxidative mechanisms (Zhang et al., 2013), these mice show greater depressive-like behavior than wild-type mice (López-Granero et al., 2017). Interestingly, increases in TNF-α, IL-1β, IL-6 and caspase-1 are found in the serum of animals submitted to chronic stress paradigms that induce depressive behavior (Zhao et al., 2019). Stress models have been used to study the etiological and developmental components of depression and to screen antidepressant drugs (Nollet et al., 2013).

Increased systemic proinflammatory cytokines can also be found in people affected with major depression before any treatment (Zou et al., 2018), but the relevance of cytokine expression in the blood of people with depressive disorders is not clear (Himmerich et al., 2019). Additionally, even though the dorsal horn of the spinal cord (DHSC) is poorly related to depression, in a model of maternal separation, there is increased microglial activation in this structure. Interestingly, the same article also showed that animals submitted to maternal separation followed by spinal nerve ligation (SNL) exhibited more relevant activation of spinal microglia (Mizoguchi et al., 2019), corroborating the hypothesis that depression may “predispose” the CNS to the pathologic properties of persistent pain. Astrocytes and microglial cells are critical for the induction and maintenance of chronic pain in a preclinical pain model (Watkins et al., 2001). These cells express Toll-like receptors (TLRs), purinergic receptors (with ATP as ligands) and glutamatergic receptors (Domercq et al., 2013; Siracusa et al., 2019). For instance, a low ATP concentration increases the release of chemokines, including IL-2, MCP-1 and CX3CL1, which act by recruiting immune cells from other brain regions or from the bloodstream to the injured tissue (Domercq et al., 2013; Turner et al., 2014). When ATP or glutamate is increased in the synaptic environment, these cells secrete cytokines, such as TNF-α, IL-1β, IL-6, and interferon (IFN)-γ (Suzuki et al., 2004; Taylor et al., 2005; Di Virgilio et al., 2009), which are pivotal inflammatory mediators that further aggravate the inflammatory reaction and may also potentially serve as biomarkers of diseases and therapeutic efficacy (Goldstein et al., 2009; Miller et al., 2009; Chen et al., 2017).

Peripheral nerve injury models present peripheral inflammation and increased proinflammatory cytokines in spinal and supraspinal structures, such as the PFC (González-Sepúlveda et al., 2016) and amygdala (Gonçalves et al., 2008) as well as with decreased thermal and mechanical nociceptive thresholds and depressive-like behavior. TNF receptor (TNFR) knockout mice are protected against CCI-induced pain; however, inflammation in the hippocampus and depressive-like behavior remain (Fischer et al., 2019). Inflammation of the limbic system also occurs in chronic stress models with increased proinflammatory cytokines in the PFC, hippocampus, raphe nuclei and pituitary (Zhao et al., 2019). Additionally, the olfactory bulbectomy-induced depression model exhibited increased CD11b (macrophage/microglia marker), GFAP (Glial fibrillary acidic protein, astrocyte marker) and IL-1β levels in the amygdala, but the spinal nerve ligation-induced neuropathic pain alone or combined with olfactory bulbectomy does not change the expression of these cytokines (Burke et al., 2013), demonstrating that although pain and depression have several similarities, they may also involve different pathways. Inflammation of the structures of the limbic system often correlates with increased serum ACTH and corticosterone levels (Zhao et al., 2019). Increased expression of these hormones is often used as a biomarker for the activation of the HPA axis, which is strongly related to stress (Abrahams et al., 2012; Stephens and Wand, 2012). Focusing on neuroinflammation, Arora and Chopra (2013) observed increases in TNF-α and IL-1β in the hippocampus and PFC after chronic administration of reserpine. In another study, chronic unpredictable mild stress and chronic corticosterone treatment induced an increase in TNF-α, IL-1β, and IL-6 in different brain structures, as well as in the hippocampus and PFC (Zhao et al., 2019). In combined models of pain and depression, increases in the cytokines IL-1β and IL-6 in the amygdala have also been noted (Burke et al., 2013). In this sense, the main general markers of the cooccurrence of chronic pain and depression are related to central inflammation, particularly TNF-α, IL-1β, and IL-6.

### Understanding Treatments for Persistent Pain and Depression

Since chronic pain and depression-pain-syndrome are complex disorders, an extensive class of medications has been used to improve the quality of life of individuals suffering from depressive-pain syndrome. Classic non-steroidal anti-inflammatory drugs (NSAIDs) and analgesics are not efficient in attenuating persistent pain sensitivity (Johannes et al., 2010); hence, the use of benzodiazepines, tricyclic antidepressants, monoamine reuptake inhibitors, glutamatergic antidepressant drugs, and adjuvant psychotherapy has been investigated to attenuate pain sensitivity. In pharmacological treatment, there is also an overlap between pain and depression: much of the medication that can be used to treat persistent pain is also used to treat major depression (Bair et al., 2003). These treatments are often related to the control of monoamines (such as serotonin, noradrenaline and dopamine), as well as glutamate and GABA, which are pivotal in the modulation of pain and depression (Benson et al., 2015). However, as described above, inflammation plays an important role in both comorbidities and, in this sense, may be a successful target for treating individuals with these symptoms.

Different types of non-pharmacological treatments can be used considering the importance of social support and the environment demonstrated in depressed subjects with chronic pain (Trief et al., 1995). Additionally, there is a strong correlation between pain, depression, and poor quality of sleep (Wilson et al., 2002; Smith and Haythornthwaite, 2004; Ohayon, 2005; Staner, 2010; Koffel et al., 2016) that is directly correlated with the level of stress. It has been shown that a stressful environment can induce hyperalgesia in rats (Rivat et al., 2007), while an enriched environment can alleviate chronic pain symptoms (Vachon et al., 2013). In addition to environmental enrichment, another non-pharmacological treatment used to investigate the inflammatory signature is pulsed radiofrequency. Non-pharmacological treatments such as acupuncture, massage and transcutaneous electrical nerve stimulation (TENS) have been administered to people affected with spinal cord injury, and they were shown to improve pain sensitivity when combined with pharmacological treatments (Norrbrink and Lundeberg, 2004). Pulsed radiofrequency uses radiofrequency to generate electrical fields that can affect neuronal membranes, altering the transmission of pain pathways (Byrd and Mackey, 2008) and is a safe and effective treatment for radicular pain, trigeminal neuralgia, occipital neuralgia, shoulder and knee pain (Vanneste et al., 2017).

NSAIDs are the most consumed drugs worldwide (Bozimowski, 2015), and their efficacy, especially in acute and postsurgical pain has been established (KuKanich et al., 2012). NSAIDs inhibit cyclooxygenase enzyme isotypes (COX1-COX4) and prostaglandins, decreasing pain, fever and inflammation by disrupting the arachidonic acid pathway (Osafo et al., 2017). This inhibition may mediate peripheral nociceptor sensitization (Vane, 1971) and the central nociceptive pathway (Hunskaar, 1987; Björkman, 1995; Wang et al., 1995). Thus, in a model of acute pain (induced by formalin) and a stressful depressive (SD) model, the effect of aspirin was compared to that of benzodiazepines and a cholecystokinin (CCK) antagonist (Rivat et al., 2010). Cholecystokinin reduces the antinociceptive effects of opioids, and its antagonist may be an effective tool to prevent opioidergic tolerance (Wiesenfeld-Hallin et al., 2002). In this article, only benzodiazepine was unable to improve the mechanical and thermal nociceptive threshold; however, aspirin was not effective in improving anxious-depressive behavior (Rivat et al., 2010), demonstrating that NSAIDs may not be helpful in the comorbidity of pain and depression comorbidity. Another class of medications used for neuropathic pain is steroidal drugs, such as corticosteroids and dexamethasone (Watanabe and Bruera, 1994). Atkinson and coworkers showed that dexamethasone is not able to control cortisol levels in individuals with chronic pain and major depression (Atkinson et al., 1986), which is related to painful and depressive symptoms (France and Krishnan, 1985). In addition, different fatty acids have been studied in an attempt to attenuate pain and depression. Omega-3 improves anxiety and depression in people affected with chronic pain (Cortes et al., 2013), while omega-3, palmatine and β-caryophyllene attenuate pain and depression in a preclinical diabetic model (Redivo et al., 2016; Shen et al., 2018; Aguilar-Ávila et al., 2019). Berberine, a quaternary ammonium salt derived from a protoberberine group of alkaloids, is the principal bioactive compound found in *Coptis chinensis*, *Berberis vulgaris* and other medicinal plants that are effective in treating pain and depression (Tang et al., 2009; Ye et al., 2009). In addition to significantly reducing hypercholesterolemia in rats (Kim and Chung, 2008; Hu and Davies, 2010; Wang et al., 2014), berberine also exerts a depressant action in the central system and has beneficial antinociceptive effects on pain threshold (Shanbhag et al., 1970). Berberine reduces pain- and depressive-like behavior in a reserpine-induced persistent pain model, and this response is related to the inhibition of SP and reactive species in the cerebral cortex and hippocampus (Arora and Chopra, 2013). Another natural compound that has been studied is triptolide, which is extracted from the medicinal plant *Tripterygium wilfordii* and can be used to suppress different proinflammatory factors from macrophages and T cells (Qiu et al., 1999; Qiu and Kao, 2003). Triptolide shows the same effectiveness in providing pain relief and attenuating depression as fluoxetine, a classical antidepressant (Hu et al., 2017).

As previously described, one of the similarities between the pathophysiology of pain and depression is the importance of monoamines. Hence, treatment with monoamine reuptake inhibitors is very common and was found in 22% of the articles that investigated treatments for pain and depression involving inflammation. Since major depression is associated with decreased serotonin levels (Haase and Brown, 2015), as in the persistent pain state (Bardin, 2011), fluoxetine improves these symptoms by increasing the amount of serotonin in the synaptic environment by selectively inhibiting the reuptake of this monoamine (Fuller et al., 1991). Although fluoxetine is the most studied selective serotonin reuptake inhibitor (SSRI) used to treat chronic pain, its use in clinical trials has been contradictory (Walker et al., 1998); however, it is effective in a preclinical lumbar disk herniation model (Cai et al., 2019). Additionally, fluoxetine plus pioglitazone or metformin, both of which are antidiabetic drugs, attenuates pain and depression with better effects than each treatment individually (Murad and Ayuob, 2015). Pioglitazone is a peroxisome proliferator-activated receptor gamma (PPARy) agonist that inhibits hyperalgesia in a spared nerve injury model (Griggs et al., 2015), while metformin inhibits mammalian target of rapamycin (mTOR) and decreases neuropathic pain and postsurgical pain (Kiałka et al., 2017). Rosiglitazone, another antidiabetic drug that also acts on PPARy, inhibits neuropathic pain induced by sciatic nerve transaction in rats by regulating macrophage polarization (Churi et al., 2008; Hasegawa-Moriyama et al., 2012). Like morphine, rosiglitazone improves mechanical allodynia, but unlike morphine, it improves depressive-like behavior by increasing BDNF and decreasing proinflammatory modulators in the hippocampus (Zong et al., 2018). Interestingly, an AMP-activated protein kinase (AMPK) inhibitor and an agonist of 3-MA (an autophagy inhibitor) block the antinociceptive, antidepressive and anti-inflammatory properties of rosiglitazone (Zong et al., 2018), suggesting that these compounds are involved in the control of pain and depression, probably due to their antidiabetic medication properties. Amitriptyline, similar to fluoxetine, is a monoamine regulator that is used regularly as an antidepressant, acts as a selective serotonin-noradrenalin reuptake inhibitor (SSNRI), and has antinociceptive effects dependent on α2-adrenergic receptors (Hiroki et al., 2017). However, in a spinal nerve ligation and olfactory bulbectomized model of pain and depression, amitriptyline treatment is not able to attenuate thermal hyperalgesia or allodynia but improves depressive-like behavior (Burke et al., 2015). According to the Cochrane Database Reviews (2014), there is not enough information to consider imipramine, which is in the same class of medicines as amitriptyline, a drug for neuropathic pain treatment. α-(Phenylselanyl)acetophenone (PSAP), a new selenium drug, has been tested for its ability to treat neuropathic pain because of its anti-inflammatory and antioxidant properties and its effect on serotonin receptors (Gerzson et al., 2012). According to Sousa et al. (2018), imipramine and PSAP improve pain sensitivity and depressive-like behavior after acute stress restriction (Sousa et al., 2018).

**FIGURE 3:**
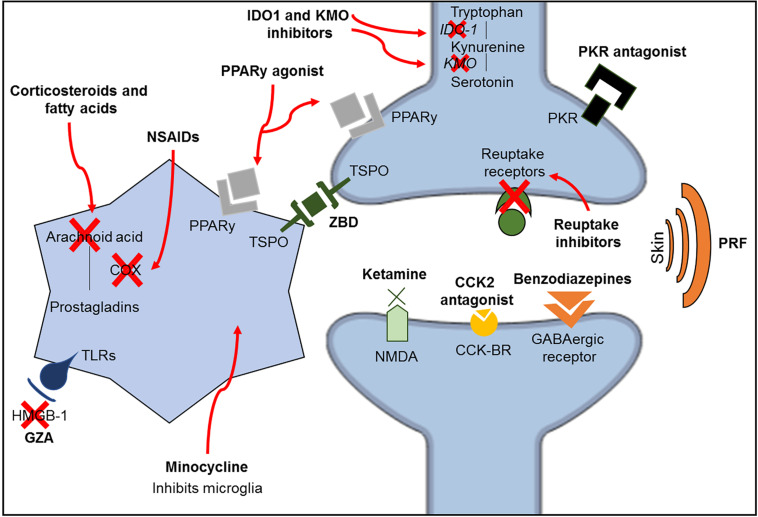
Schematic representation of the classic mechanisms of the different classes of medications investigated in this review. CCK2, cholecystokinin 2; CCK-R, cholecystokinin receptors; GZA, glycyrrhizic acid; HMGB-1, high mobility group box-1; IDO1, indolamine 1,3 deoxygenase; KMO, kynurenine 3-monooxygenase; NMDA, *N*-methyl-D-aspartate; NSAIDs, non-steroidal anti-inflammatory drugs; PKR, prokineticin family; PPARy, peroxisome proliferator-activated receptor gamma; PRF, pulsed radiofrequency; TLRs, toll-like receptors; TSPO, translocator protein; ZBD, *N*-benzyl-Nethyl-2-(7,8-dihydro-7-benzyl-8-oxo-2-phenyl-9H-purin-9-yl) acetamide.

Ketamine is an anesthetic drug with *N*-methyl-D-aspartate (NMDA) receptor antagonism properties. Since these receptors are related to the amplification of pain signals and opioid tolerance, ketamine acts by reducing these dysfunctions observed in chronic pain conditions (Trujillo and Akil, 1991; Laulin et al., 2002; Lilius et al., 2015). Additionally, ketamine may also regulate monoaminergic receptors (Peltoniemi et al., 2016) and increase the inhibitory serotonergic pathway (Mion and Villevieille, 2013). However, ketamine has been shown to improve depressive-like behavior without attenuating pain in a spared nerve injury model (Pan et al., 2018). Another possible antinociceptive mechanism of ketamine is the inhibition of astrocyte and microglia activation (Sleigh et al., 2014), as minocycline, an antibiotic with high lipid solubility, crosses the BBB and inhibits microglia activation (Yrjänheikki et al., 1998). Minocycline improves pain sensitivity and depressive-like behavior in a model of spinal nerve ligation combined with olfactory bulbectomy (Burke et al., 2014); however, it was shown to be ineffective in patients with lumbar radicular neuropathic pain in a double-blind clinical trial using amitriptyline as a comparator (Vanelderen et al., 2015). A summary of the pharmacological mechanisms of the treatments described in this review is shown in Figure 3 and Table 2.

**TABLE 2:** Inflammatory response regarding different types of treatments.

Pain models investigating depression						
References		Pain model	*Therapeutic intervention*	After treatment		
				Painful behavior	Depressive-like behavior	Inflammatory effects
Birmann et al., 2019		PSNL	*3-(4-Chlorophenylselany)-1-methyl-1H-indole*	↑ Thermal nociceptive threshold using the hot plate test	↓ Immobility in the FST	↓ ROS and lipid peroxidation in the cortex and hippocampus
				↑ Mechanical nociceptive threshold using the von Frey test	↑ Grooming in the splash test	↓ Costicosterone in plasma

Zhang et al., 2016		CFA	*Acute dose of ketamine*	↑ Thermal nociceptive threshold using the hot plate test	↓ Immobility in the FST	↓ IL-6 and IL-1β in the hippocampus
				↑ Mechanical nociceptive threshold using the von Frey and paw pressure tests	↑ Sucrose preference	↓ Indoleamine 2,3-dioxygenase and kynurenine in the hippocampus

Vachon et al., 2013		SNI	*Environmental enrichment*	↑ Thermal nociceptive threshold using the hot plate and acetone tests	No change in time in close-arms in the EPM	↓ SP and CGRP in the lumbar spinal cord
				↑ Mechanical nociceptive threshold using the von Frey test	No change in time in the tail suspension	

Murad and Ayuob, 2015		CCI	*Fluoxetine*	↑ Mechanical nociceptive threshold using the von Frey test	↓ Immobility in the FST	↓ TNF-α, IL-6, MCP-1 in plasma
						↑ IL-10 in plasma
						↓ GFAP expression in the spinal cord
						↓ Myelin degeneration and leukocyte infiltration
	
			*Pioglitazone*	↑ Thermal nociceptive threshold using the Hargreaves test	↓ Immobility in the FST	↓ TNF-a, IL-6, MCP-1 in plasma
						↑ IL-10 in plasma
				↑ Mechanical nociceptive threshold using the von Frey test		↓ GFAP expression in the spinal cord
						↓ Myelin degeneration and leukocyte infiltration
	
			*Metformin*	↑ Thermal nociceptive threshold using the Hargreaves test	↓ Immobility in the FST	↓ TNF-α, IL-6, MCP-1 in plasma
						↑ IL-10 in plasma
				↑ Mechanical nociceptive threshold using the von Frey test		↓↓ GFAP expression in the spinal cord
						↓ Myelin degeneration and leukocyte infiltration
	
			*Fluoxetine + pioglitazone*	↑ Thermal nociceptive threshold using the Hargreaves test	↓↓ Immobility in the FST	↓ TNF-α, IL-6, MCP-1 in plasma
						↑ IL-10 in plasma
				↑ Mechanical nociceptive threshold using the von Frey test		↓↓ GFAP expression in the spinal cord
						↓↑ Myelin degeneration and leukocyte infiltration
	
			Fluoxetine + metformin	↑ Thermal nociceptive threshold using the Hargreaves test	↓ Immobility in the FST	↓ TNF-α, IL-6, MCP-1 in plasma
						↑ IL-10 in plasma
				↑ Mechanical nociceptive threshold using the von Frey test		↓↓ GFAP expression in the spinal cord
						↓↓ Myelin degeneration and leukocyte infiltration

Cai et al., 2019		LDH	*Fluoxetine*	↑ Thermal nociceptive threshold using the Hargreaves test	↓ Immobility in the FST	↓ TNF-α in the hippocampus
				↑ Mechanical nociceptive threshold using the von Frey test	↑ Sucrose preference	

Fang et al., 2019		SNI	*IRF8 siRNA*	↑ Mechanical nociceptive threshold using the von Frey test	↓ Immobility in the FST	↓ IRF8 in the spinal cord
					↑ Sucrose preference	↓ BDNF in the NAc
	
			Pulsed frequency	↑ Mechanical nociceptive threshold using the von Frey test	↓ Immobility in the FST	↓ IRF8 in the spinal cord
					↑ Sucrose preference	↓ BDNF in the NAc
Redivo et al., 2016		STZ	*Fish oil*	↑ Mechanical nociceptive threshold using the von Frey test	↓ Immobility in the FST (modified)	↑ BDNF in the hippocampus and PFC

Li et al., 2017		SCI	*ZBD-2*	↑ Thermal nociceptive threshold using the Hargreaves test	↓ Immobility in the FST	↓ Iba-1 and GFAP expression in the hippocampus and spinal cord
				↑ Mechanical nociceptive threshold using the von Frey test	↓ Immobility in the tail suspension	↑ BDNF in the hippocampus and spinal cord
						↓ Costicosterone in plasma

Shen et al., 2018		STZ	*Palmatine*	↑ Thermal nociceptive threshold using the Hargreaves test	↓ Immobility in the FST	↓ GFAP and P2X7 expression in the hippocampus
				↑ Mechanical nociceptive threshold using the von Frey test	↑ Sucrose preference	↓ TNF-α and IL-1β in the hippocampus

Hisaoka-Nakashima et al., 2019		PSNL	*GZA (anti HMGB-1)*	Not measure	↓ Immobility in the FST	↓ Iba-1 expression (activation) in the PFC
					↑Social interaction	
					↓ Novelty suppressed feeding	

Li et al., 2019		CCI	*AcYVAD-CMK (caspase-1 inhibitor)*	No change in thermal nociceptive threshold using the Hargreaves test	↓ Immobility in the FST	Not measure
				No change in mechanical nociceptive threshold using the von Frey test	↑ Climbing in the FST No change in the EPM	
	
			*2-AP (2-aminopurine - PKR inhibitor)*	No change in thermal nociceptive threshold using the Hargreaves test	↓ Immobility in the FST	↓ Inflamassome (NLRP1) and caspase-1 expression in the hippocampus
					No change in mechanical nociceptive threshold using the von Frey test	↑ Climbing in the FST No change in the EPM	

Pan et al., 2018		SNI	*Ketamine*	No change in mechanical nociceptive threshold using the von Frey test	↓ Immobility in the FST	↑ BDNF in the PFC
						↑ NL1 and ↓ NL2 in the PFC
						↑ BDNF in the ACC
						No change in NL1 and NL2 in the ACC
						↑ BDNF in the hippocampus
						No change in NL1 and ↑ NL2 in the hippocampus

Brüning et al., 2015		PSNL	*acute (m-CF3-PhSe)2*	↑ Mechanical nociceptive threshold using the von Frey test	↓ Immobility in the FST	No change in pro-inflammatory cytokines in serum, cortex and hippocampus
						↓ COX2 and NF-κB in the cortex
						↑ BDNF in cortex
						↓ COX2 and no change in NF-κB in the cortex
						↑ BDNF in the hippocampus
						No change in ACTH and corticosterone
	
			*subchronic (m-CF3-PhSe)2*	↑ Mechanical nociceptive threshold using the von Frey test	↓ Immobility in the FST	↓ IL-1β, IL-6, TNF-α, IFN-γ and ↑ IL-10 in serum
						↓ IL-1β, IL-6, TNF-α, IFN-γ in the cortex and hippocampus
						↓ COX2 and NF-κB in the cortex
						↑ BDNF in cortex
						↓ COX2 and no change in NF-κB in the hippocampus
						↑ BDNF in the hippocampus
						↓ ACTH and corticosterone in serum

Zhou et al., 2015		SNI	*IL-1RA*	↑ Mechanical nociceptive threshold using the von Frey test	↓ Immobility in the FST	↓ Ido-1 and IL-1β in liver
	
			*Ido -/-*	No change in mechanical nociceptive threshold using the von Frey test	↓ Immobility in the FST ↑ Social interaction	Not measure

Laumet et al., 2018		SNI	*IL-1RA inhibitor*	No change in mechanical nociceptive threshold using the von Frey test	↓ Immobility in the FST	Not measure
	
			*Ro61-8048 (KMO inhibitor)*	No change in mechanical nociceptive threshold using the von Frey test	↓ Immobility in the FST	Not measure

Hu et al., 2017		SNL	*Triptolide*	↑ Mechanical nociceptive threshold using the von Frey test	↓ Immobility in the FST	↓ Iba-1 and p38 expression in the hippocampus
					↑ Sucrose preference	↓ IL-1β, TNF-α and ↑ IL-10 in the hippocampus
	
			*Fluoxetine*	↑ Mechanical nociceptive threshold using the von Frey test	↓ Immobility in the FST	↓ Iba-1 and p38 expression in the hippocampus
					↑ Sucrose preference	No change in IL-1β and TNF-α; ↑ IL-10 in the hippocampus
	
			*Fluoxetine + triptolide*	↑ Mechanical nociceptive threshold using the von Frey test	↓ Immobility in the FST	↓ Iba-1 and p38 expression in the hippocampus
					↑ Sucrose preference	↓ IL-1β, TNF-α and ↑ IL-10 in the hippocampus

Moschetti et al., 2019		Vincristine	*PC1 (PK-Rs antagonist)*	↑ Thermal nociceptive threshold using the plantar and acetone tests	No difference in the FST and sucrose preference	↓ IL-1β, TNF-α, IL-6, TLR4, CD68 and CD11b and ↑ IL-10 in the DRG
				↑ Mechanical nociceptive threshold using the von Frey test		↓ IL-1β, TNF-α, TLR4, CD68 and CD11b and ↑ GFAP in the spinal cord

Aguilar-Ávila et al., 2019		STZ	*β-Caryophyllene*	↑ Thermal nociceptive threshold using the hot-plate test	↓ Immobility in the FST	↓ SP, IL-1β, TNF-α and IL-6 in serum
				↑ Mechanical nociceptive threshold using the von Frey test and SMALGO^®^	↓ Immobility in the tail suspension ↓ Murbles touched	

Arora and Chopra, 2013		Reserpine	*Berberine*	↑ Thermal nociceptive threshold using the tail-immersion test	↓ Immobility in the FST	↓ SP in the cortex and hippocampus
				↑ Mechanical nociceptive threshold using the paw pressure and von Frey tests		↓ Lipid peroxide, non-protein thiols, superoxide dismutase and nitrite levels in the cortex
						↓ Lipid peroxide, non-protein thiols, superoxide dismutase and nitrite levels in the hippocampus

Zong et al., 2018		SNT	*Rosiglitazone*	↑ Mechanical nociceptive threshold using the von Frey test	↓ Immobility in the FST	↑ BDNF in HC
					↑ Sucrose preference	↓ TNF-α, IL-1β, SOD e MDA in the hippocampus
					↓ Immobility in the tail suspension	
	
			*Morphine*	↑ Mechanical nociceptive threshold using the von Frey test	No change in immobility in the FST	Not measure
					No change in the sucrose preference	
					No change in immobility in the tail suspension	
	
			*Rosiglitazone + Compound C (AMPK inhibitor)*	No change in mechanical nociceptive threshold using the von Frey test	No change in immobility in the FST	↓ TNF-α, IL-1β, SOD e MDA in the hippocampus
					No change in the sucrose preference	
					No change in immobility in the tail suspension	
	
			*Rosiglitazone + 3-methyladenine (3-MA - autophagic antagonist)*	No change in mechanical nociceptive threshold using the von Frey test	No change in immobility in the FST	No change in TNF-a, IL-1b, SOD e MDA in the hippocampus
					No change in the sucrose preference	
					No change in immobility in the tail suspension	

**Depressive-like models investigating pain**						

**References**		**Depressive-like model**	***Therapeutic intervention***	**After treatment**		
				**Painful behavior**	**Depressive-like behavior**	**Inflammatory effects**

Sousa et al., 2018		Acute stress restriction	*α-(phenylselanyl) acetophenone (PSAP)*	↑ Thermal nociceptive threshold using the hot plate test	↓ Immobility in the FST	↓ Lipid peroxidation, reactive species, nitrite and nitrate in the cortex
					↑ Sucrose preference	↓ Lipid peroxidation, reactive species, nitrite and nitrate in the hippocampus
				↑ Mechanical nociceptive threshold using the von Frey	↑ Time spent in open arms in the EPM	↓ Costicosterone in plasma
					↓ Murbles buried	
	
			*Imipramine*	↑ Thermal nociceptive threshold using the hot plate test	↓ Immobility in the FST	↓ Lipid peroxidation, reactive species, nitrite and nitrate in the cortex
					↑ Sucrose preference	↓ Lipid peroxidation, reactive species, nitrite and nitrate in the hippocampus
				↑ Mechanical nociceptive threshold using the von Frey test	↑ Time spent in open arms in the EPM	↓ Costicosterone in plasma
					↓ Murbles buried	

**Pain and depressive-like models**						
**References**	**Pain model**	**Depressive-like model**	**Therapeutic intervention**	**After treatment**		
				**Painful behavior**	**Depressive-like behavior**	**Inflammatory effects**

Burke et al., 2015	SNL	OB	Amitriptyline	No change in thermal nociceptive threshold using the Hargreaves and acetone tests	↓ Noxious avoidance behavior	↓ GFAP expression in the PFC
				↑ Mechanical nociceptive threshold using the von Frey test		↓ IL-10 and CCL5 and ↑ TNF-α in the PFC

Rivat et al., 2010	Formaline	Social defeat	Chlordiazepoxide	No change in mechanical nociceptive threshold using the von Frey and paw pressure tests	↑ Time spent in the open arms in the EPM	↓ iNOS and COX2 in the PFC
				No change in nociceptive threshold by Dubusson and Dennis score		
			
			Aspirine	↑ Mechanical nociceptive threshold using the von Frey and paw pressure tests	No change in the EPM	↓ iNOS and COX2 in the PFC
				↑ Nociceptive threshold by Dubusson and Dennis score		
			
			CCK2 antagonist (i.p)	↑ Mechanical nociceptive threshold using the von Frey and paw pressure tests	↑ Time spent in the open arms in the EPM	↓ iNOS and COX2 in the PFC
				↑ Nociceptive threshold by Dubusson and Dennis score		
			
			CCK2 antagonist (RVM injection)	↑ Mechanical nociceptive threshold using the von Frey and paw pressure tests	Not measure	Not measure
				↑ Nociceptive threshold by Dubusson and Dennis score		

Burke et al., 2014	SNL	OB	Mynocyclin	↑ Thermal nociceptive threshold using the acetone test	Locomotor hyperactivity in the OFT	↑ IL-1β, IL-6 and SOCS3 in the PFC
				↑ Mechanical nociceptive threshold using the von Frey test		↑ IL-10 and MRC2 in the PFC

**Clinical data**						
**References**		**Diagnosis**	**Therapeutic intervention**	**After treatment**		
				**Painful behavior**	**Depressive-like behavior**	**Inflammatory effects**

Atkinson et al., 1986		Chronic pain with coexisting major depression	Dexamethasone	Not measure	Not measure	Non-suppressed cortisone levels

Wingenfeld et al., 2010		Fibromyalgia	DEX and DST	Pain Experience Scale Heat and pressure Pain	SCID-II BDI	MDD patients ↑ cortisol before or after DEX
						Patients with fibromyalgia + MDD ↑ cortisol
						↑ depression x ↑ sensitivity to pressure pain

France and Krishnan, 1985		Chronic low back	DEX and DST	Not measure	DSM- III	↑ cortisol in chronic back patients with major depression (related with depressive symptoms)

*BDI-II, Beck Depression Inventory; BDNF, brain-derived neurotrophic factor; CCI, chronic constriction injury; CD11b, cluster of differentiation 11b; CD68, cluster of differentiation 68; CFA, complete Freund’s adjuvant; CGRP, calcitonin gene related peptide; COX2, cyclooxygenase 2; DEX, dexamethasone; DSM-III, Diagnostic and Statistical Manual of Mental Disorders III; DST, suppression test; EPM, elevated plus maze; FST, forced swimming test; GFAP, glial fibrillary acidic protein; HC, hippocampus; Iba-1, ionized calcium-binding adapter molecule 1; IL, interleukin; IL-1RA, interleukin 1 receptor antagonist; IRF8, interferon regulatory factor 8; Ido, indoleamine 2,3, dioxygenase inhibitor; iNOS, inducible nitric oxide synthase; LDH, lumbar disk herniation; MCP1, monocyte chemoattractant protein-1; MDA, malondialdehyde; MDD, major depressive disorder; MRC2, mannose receptor C type 2; NF-κB, factor nuclear kappa B; NL1, neuroligins 1; NL2, neuroligins 2; NLRP-1 inflamassome, Nod-like receptor protein (NLRP)-1 inflammasome; OB, olfactory bulbectomy; P2X7, purinergic receptor P2X7; p38, p38 mitogen-activated protein kinases – MAPKs; PC1, triazine guanidine derivative selective; PFC, prefrontal cortex; PSNL, partial spinal nerve ligation; SCI, spinal cord injury; SCID-II, Structured Clinical Interview for DSM-IV Axis II Personality Disorders; siRNA, small interfering RNA; SNI, spared nerve injury; SNL, spinal nerve ligation; SNT, sciatic nerve transaction; SOCS3, suppressor of cytokine signaling 3; SOD, superoxide dismutase; SP, substance P; STZ, streptozotocin; TNF-α, tumor necrosis factor alfa.*

Because of the complexity and multifactorial aspects of persistent pain and depression, an extensive number of medications are already used by individuals suffering from depression-pain syndrome but are often not effective. Hence, it is important to investigate new mechanisms of classical drugs to improve their use and to find and develop new drugs and targets to treat these syndromes.

### Understanding the Anti-inflammatory Signature of Depressive-Pain Treatments

Beyond understanding the neuroinflammatory signature of the pathophysiology of pain and depression, we found a total of 23 articles (39%) that investigated the role of different types of treatments in controlling pain, depressive-like behavior and neuroinflammation. We will address the treatments used in the articles found in our research, the identified neuroinflammatory signatures in response to treatment and the possible neuronal networks involved in the amelioration of depressive-pain symptoms.

In preclinical trials, it is common to investigate molecular inhibitors or antagonists in an attempt to understand the specific role of different neuromodulators. We found that in 39% of the preclinical models, treatments involving different inhibitors or antagonists were investigated. Acute or subchronic treatment with these inhibitors may shed light on the possible mechanisms of pain and depression or may indicate a new target for treating these syndromes.

Neuroimaging and postmortem studies have shown an increase in mitochondrial translocator protein (tryptophan-rich sensory proteins -TSPO) in subjects with major depressive disorder (Enache et al., 2019). It has been shown that during local or systemic inflammation, TSPO is overexpressed in inflammatory cells and can be considered a marker of proinflammatory microglia (Selvaraj and Tu, 2016; Owen et al., 2017; Beckers et al., 2018). Li et al. (2017) showed that *N*-benzyl-*N*-ethyl-2-(7,8-dihydro-7-benzyl-8-oxo-2-phenyl-9H-purin-9-yl) acetamide (ZBD-2), a TSPO ligand, decreases microglia and astrocyte activation and increases BDNF expression in the hippocampus and spinal cord in neuropathic rats subjected to spinal cord injury, improving pain sensitivity and depressive-like behavior by decreasing corticosterone levels in the plasma. This finding corroborates the idea that mitochondrial dysfunction plays a pivotal role in inflammation and DAMP formation (Shimada et al., 2012).

Different types of inhibitors of the inflammatory cascade have been used to attenuate painful and depressive-like behavior. The prokineticin family receptor (PKR) is present in the DRG and spinal cord and plays a role in sensitizing nociceptors via transient receptor potential vanilloid receptor 1 (TRPV1), inducing the release of proinflammatory cytokines (Negri et al., 2006; Li et al., 2016). PC-1, a PKR antagonist, was used in a model of chemotherapy-induced neuropathic pain with vincristine and was shown to decrease the levels of proinflammatory cytokines and infiltrating leucocytes without changing the expression of GFAP in the spinal cord (Moschetti et al., 2019), demonstrating that the antinociceptive properties of PKR inhibition may be related to peripheral mechanisms. Reinforcing these results, 2-AP, another PKR inhibitor, was also shown to decrease inflammasome and caspase-1 expression in the hippocampus of rats subjected to chronic constriction injury (Li et al., 2019). The tryptophan-metabolizing enzyme indolamine 1,3 deoxygenase (IDO1) is a direct modulator of inflammatory cytokine release (O’Connor et al., 2009) and is pivotal for the metabolism of tryptophan into kynurenine (KIN), which is further converted to kynurenine 3-monooxygenase (KMO) (Schwarcz and Stone, 2017). In this sense, the effect of inhibition of IDO and KMO, which attenuates depressive-like behavior but weakly ameliorates painful behavior, was compared to that of an inhibitor of IL-1 receptor antagonist (IL-1RA) in two different articles (Zhou et al., 2015; Laumet et al., 2017).

As previously described, glial cells are very important for the maintenance of the inflammatory response within the nervous system. Interferon regulatory factor 8 (IRF8) deficiency prevents the activation of microglia (Liu et al., 2018), and the mechanisms by which IRF8 deficiency improves pain sensitivity and depressive-like behavior are very similar to those of pulsed radiofrequency, which decreases IRF8 in the spinal cord and BDNF in the NAc, demonstrating that non-pharmacological treatments also play an important role in microglial activation and the VTA-NAc reward pathway (Fang et al., 2019). High-mobility group box-1 (HMBG1) is secreted and activates immune cells via Toll-like receptor 4 (TLR4), inducing the production of cytokines and chemokines (Andersson and Tracey, 2011; Agalave and Svensson, 2015). Since HMBG1 is upregulated in the spinal cord and sciatic nerve in neuropathic pain models (Ren et al., 2012; Nakamura et al., 2013), the effect of glycyrrhizic acid (GZA), an inhibitor of HMGB-1, in a partial sciatic nerve ligation model was investigated. GZA attenuates depressive-like behaviors and decreases microglial activation in the PFC (Hisaoka-Nakashima et al., 2019). In relation to microglia and depressive-pain syndrome, minocycline, which also inhibits microglial activation, normalizes thermal and mechanical nociceptive thresholds and improves anxiety-depressive behavior (Burke et al., 2014; Xu et al., 2017; Dai et al., 2019). Additionally, similar to macrophages, microglia is also polarized toward the classical proinflammatory M1 phenotype or the alternative anti-inflammatory M2 phenotype (Jha et al., 2016). Although minocycline is able to increase the expression of IL-10 and mannose receptor (MRC2), both of which are markers of the M2 phenotype, it does not inhibit the increases in IL-1β, IL-6 or suppressor of cytokine signaling 3 (SOCS3) in the PFC in a spinal nerve ligation and olfactory bulbectomy model (Burke et al., 2014).

The compound *m*-trifluoromethyl-diphenyl diselenide (*m*-CF3-PhSe)2 permeates the BBB and modulates opioid receptors without inducing opioid tolerance (Brüning et al., 2015; Rosa et al., 2018). Acute and subchronic treatment with this compound improves mechanical nociceptive thresholds and depressive-like behavior, decreases COX-2 and increases BDNF in the cerebral cortex and hippocampus in a partial sciatic nerve ligation model; however, only subchronic administration decreases proinflammatory cytokines and increases IL-10 in the serum, cerebral cortex and hippocampus and decreases corticosterone in the serum (Brüning et al., 2015). Although aspirin, a classic NSAID, is not able to improve depressive-like behavior, like benzodiazepines and a cholecystokinin 2 (CCK2) antagonist, it decreases Inducible nitric oxide synthase (iNOS) and COX2 in the PFC in the formalin test and a social defeat model (Rivat et al., 2010). Fatty acids with anti-inflammatory properties, such as omega-3, which increases BDNF in the hippocampus and PFC (Redivo et al., 2016); palmatine, which decreases GFAP and purinergic receptor P2X7 expression in the hippocampus (Shen et al., 2018); and *b*-caryophyllene, which suppresses the levels of SP and proinflammatory cytokines in the serum of diabetic animals (Aguilar-Ávila et al., 2019), may be used to attenuate pain and depression. Additionally, like fluoxetine, triptolide decreases microglial activation in the hippocampus, but only triptolide or triptolide combined with fluoxetine decreases proinflammatory cytokines and increases IL-10 in the hippocampus after improving spinal nerve ligation-induced pain and depressive behavior (Hu et al., 2017).

Indeed, manipulation of monoamines may also involve anti-inflammatory mechanisms. In a lumbar disk herniation model, fluoxetine decreases TNF-α in the hippocampus (Cai et al., 2019), while in a chronic constriction injury model, it decreases proinflammatory cytokines in the plasma, increases IL-10, decreases myelin degeneration and leukocyte infiltration in peripheral nerves and decreases astrocyte activation in the spinal cord; additionally, it has better effects when combined with antidiabetic drugs (Murad and Ayuob, 2015). Despite having similar mechanisms, amitriptyline does not improve thermal nociceptive thresholds but decreases astrocyte activation (Burke et al., 2015). Interestingly, this decrease is accompanied by decreased IL-10 and C-C chemokine ligand 5 (CCL5/RANTES) but increased TNF-α in the PFC in a co-model of pain and depression induced by spinal nerve ligation and olfactory bulbectomy model (Burke et al., 2015). A possible explanation for this finding is that CCL5 is released upon TNF-α activation to regulate microglia-astrocyte crosstalk and glutamate reuptake in the PFC (Pittaluga, 2017). The antioxidant α-(phenylselanyl) acetophenone (PSAP) was compared to imipramine, another drug that modulates monoamines. PSAP also seems to interact with serotonin type 1A (5HT-1A) receptor but not with noradrenaline, dopamine or adenosine receptors (Gerzson et al., 2012). In addition to improving behavior, both drugs decrease lipid peroxidation, reactive oxygen species (ROS), nitrite and nitrate levels in the cerebral cortex and hippocampus and inhibit corticosterone levels in the plasma after acute stress restriction (Sousa et al., 2018). Another new compound with monoaminergic properties is 3-(4-chlorphenylselany)-1-methyl-1H-indole, which improves pain sensitivity and depressive-like behavior and, like PSAP and imipramine, also decreases ROS and lipid peroxidation in the cerebral cortex and hippocampus and corticosterone in the plasma (Birmann et al., 2019). In this sense, the regulation of monoamines is clearly pivotal to the control of neuroinflammation. Possibly, improvements in the synaptic environment by the normalization of neurotransmitter levels decreases DAMPs, such as ROS and reactive nitrogen species (NOS), and inhibits the activation of glial cells, decreasing the release of proinflammatory cytokines. Persistent activation of NMDA receptors by glutamate cytotoxicity also plays an important role in DAMP formation and, consequently, in the pathophysiology of pain and depression (Petrenko et al., 2003; Duman and Li, 2012). Acute ketamine decreases proinflammatory cytokines, IDO1 and kynurenine in the hippocampus after improving painful and depressive behavior in a model of complete Freund’s adjuvant-induced acute pain (Zhang et al., 2016). However, in a persistent pain model, such as a sciatic nerve injury model, ketamine decreases depressive-like behavior and increases BDNF in the PFC and ACC but does not change the mechanical nociceptive threshold (Pan et al., 2018). These findings corroborate the strong evidence that ketamine decreases postoperative pain scores in acute nociceptive pain (Bell and Kalso, 2018) but that it seems to be less effective for chronic pain treatment. However, the central mechanism by which ketamine exerts neuroprotection in the PFC and ACC suggests that it is useful for treating major depression disorder (Daly et al., 2018).

### Role of Inflammation in Neuronal Networks

Microglia is the most abundant resident immune cell in the CNS, able to recognize DAMPs and pathogen-associated molecular patterns (PAMPs) and respond by producing many inflammatory mediators (Dheen et al., 2007). In addition to being important for the immune response, microglia also play a pivotal role in the pruning of synaptic spines induced by sensory inputs or neuronal injury (Wake et al., 2009; Tremblay et al., 2010). In this sense, microglia-deficient brain slices show an increase in excitatory synapses, while the presence of microglia suppresses the AMPA receptor within the neuronal membrane and synaptic adhesion molecules (Ji K. et al., 2013). In addition, one astrocyte may interact with over 100,000 synapses to form a tripartite synapse (Halassa et al., 2007; Araque, 2008). Astrocytes can coordinate and control synaptic transmission through neurotransmitter receptors, transporters, and cell adhesion molecules (Halassa et al., 2007). The cytotoxicity induced by excessive glutamate in the synaptic environment is prevented by the astrocyte reuptake process (Hertz and Zielke, 2004), suggesting the pivotal role of these cells in the control of synapses.

In pathological conditions after an injury or disease, microglial cells may show the classical activated pattern (M1) characterized by upregulation of nuclear factor-κB (NF-κB), signal transducer and activator of transcription (STAT), TNF-α, IL-1β and different proinflammatory mediators (Jha et al., 2016). M1 microglia have the capacity to induce changes in the phenotype of astrocytes to the classical activation phenotype (A1), which is consistent with the increase in inflammation and tissue damage (Liddelow et al., 2017) seen in depressive pain syndrome. A1 astrocytes decrease the formation of excitatory synapses in an *in vitro* coculture system and decrease the frequency and amplitude of excitatory postsynaptic currents in the remaining synapses (Liddelow et al., 2017). Indeed, pain and depression conditions induce the downregulation of glutamate type 1 transporter (GLT1) in response to inflammation (Medina et al., 2013; Guo et al., 2019), suggesting that the astrocyte-reuptake process involved in pruning synapses is impaired in depression-pain syndrome. As described above, the cooccurrence of chronic pain and depression is intimately related to increases in TNF-α, IL-1β, and IL-6 in target structures such as the PFC, ACC, amygdala and hippocampus, emphasizing the involvement of glial activation in limbic regions involved in pain and depression processing.

**FIGURE 4:**
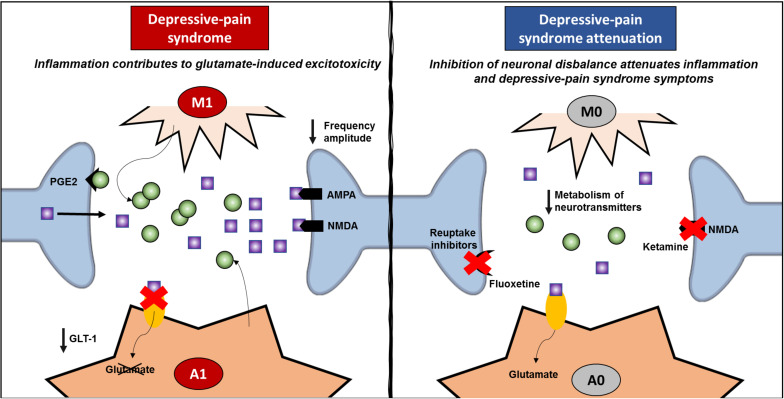
Review of inflammatory signature and neural network modulation. Depressive pain syndrome is related to activation of glial cells, such as microglia and astrocytes. Once classically activated (M1/A1), these cells release proinflammatory cytokines, especially TNF-α and IL-1β, and upon specific receptor activation, increase glutamate release in the synaptic cleft. Classically activated astrocytes induce a decrease in GLT-1, which enables the reuptake of glutamate and decreases glutamate-induced cytotoxicity. Hence, both AMPA and NMDA postsynaptic receptors are strongly activated, decreasing the frequency amplitude and contributing to neural network imbalance. On the other hand, anti-inflammatory treatments inhibit the classic activation of glial cells and the exacerbated release of proinflammatory mediators, decreasing neural network imbalance. In addition, the success of pharmacological treatments that act through mechanisms related to neuronal dysfunction, such as ketamine and fluoxetine, which improve neuronal network dysfunction and attenuating depressive pain syndrome, also corroborate the negative feedback of inflammation. GLT, glutamate transporter; PGE, prostaglandin.

Even though IL-1β is related to resident glial cells, it has been shown that this cytokine plays a role in synaptic plasticity (Kelly et al., 2003; Mandolesi et al., 2013). Once recognized by neurons, IL-1 increases glutamate release through prostaglandin (PGE)-2 production (Sang et al., 2005; Mishra et al., 2012), activating NMDA receptors, increasing glutamatergic sensitivity and exacerbating excitotoxicity (Viviani et al., 2006; Fogal and Hewett, 2008). In the rat hippocampus, IL-1β decreases synaptic connections by increasing both pre- and postsynaptic glutamate release (Mishra et al., 2012). In this sense, inflammation inhibits the ability of astrocytes to reuptake glutamate, and IL1-β increases glutamate levels within the synaptic cleft. TNF-α also plays a role in increasing the exocytosis of AMPA receptors on hippocampal pyramidal cells (Ogoshi et al., 2005) while promoting the endocytosis of GABA A receptors (Stellwagen et al., 2005), triggering an imbalance between excitatory and inhibitory controls. The relationship between inflammation and the neuronal environment investigated by two articles is described in Table 1. González-Sepúlveda et al. (2016) showed that the increase in IL-1β in the PFC relates to an increase in the glutamate/glycine ratio, while Dellarole et al. (2014) demonstrated that TNFR-deficient mice are protected against increases in neurogenesis, neuroplasticity and myelin impairments induced by neuropathic pain. TNF-α also inhibits noradrenaline release in the hippocampus (Spengler et al., 2007), which is postulated as a common target in pain and depression (Fasick et al., 2015). Hence, inhibiting inflammation may be an important tool for restarting the optimal modulation and control of synapses within the CNS, improving depressive pain symptoms. However, ketamine, which acts on NMDA receptors, not only decreases inflammatory markers, as previously described but also inhibits the KYN/tryptophan ratio while increasing the 5-HT/tryptophan ratio (Zhang et al., 2016). Accordingly, fluoxetine increases 5-HT in the hippocampus while decreasing inflammation (Cai et al., 2019), suggesting that improving the synaptic network also affects inflammation status (Figure 4). The same rationale may apply to other treatments, such as benzodiazepines, reuptake inhibitors, and CCK2 and PKR inhibitors, which classically involve pre- and postsynaptic neurons, as described above. In addition to acting through a neuronal mechanism, these drugs are able to inhibit inflammation in depressive-pain models.

Taken together, our findings suggest that neuronal deficits and the inflammatory response may trigger each other through a feedback mechanism, contributing to the complexity of the control of depression-pain syndrome. In this sense, it is important for treatments to address the role of neurotransmitters and receptors as well as to improve the entire synaptic cleft, including inhibiting classically activated resident cells and the inflammatory response. In other words, these data strongly support the idea that controlling neuroinflammation is closely connected with improving pain and depression states. Direct inhibition of the inflammatory cascade or indirect inhibition by decreasing cytotoxicity and DAMP formation in local synapses concomitant with controlling glial cell activation are important strategies for improving the quality of life of people suffering from depression-pain syndrome.

## Conclusion and Future Directions

Pain and depression are often comorbid and decrease the quality of life of many individuals worldwide. Indeed, much effort has been expended to investigate different classes of medications to ameliorate these symptoms. In this sense, preclinical models are a resource for the development of drugs or for better elucidating their peripheral and central mechanisms. It is important to take into consideration that preclinical models are tools to provide a better understanding of pathogenesis and for testing the potential of novel therapeutic approaches. However, there are limitations intrinsic from the experimental models referring to translate research into practice. The choice of the appropriate preclinical model to address pharmacological treatments should be consider as the most reliable and closer to reproduce a persistent pain as observed in clinical practice. Even though the models should be able to reproduce the nociceptive behavior, it is pivotal that they also reproduce depressive-anxiety behavior, mimicking what is observed in patients. Additionally, the experimental design should be carefully addressed taking into consideration the presence of peripheral and central sensibilization before the initiation of the pharmacological treatment as it happens with individuals suffering from persistent pain. In addition to directly targeting inflammation mediators such as cytokines and molecular inhibitors of the inflammatory cascade to improve pain sensitivity and depression, drugs that improve and regulate the synaptic environment and its neurotransmitters may act as anti-inflammatory compounds, reducing local DAMPs and inhibiting the activation of immune and glial cells. In this review, we highlight the inflammatory signature of different preclinical models and clinical articles related to depressive-pain syndrome. Additionally, we discuss the role of therapeutic interventions targeting depression, pain and neuroinflammation. We hope that shedding light on the inflammatory profile will aid in finding new targets and in improving classic treatments to bring benefits to individuals who suffer from these disorders.

## Author Contributions

AC and GA contributed to the conception of the study, acquisition of the data, and writing of the manuscript. MM and RP wrote and revised the manuscript. RM conceived the presented review, supervised the project, and wrote the manuscript. All the authors contributed to the article and approved the submitted version.

## Conflict of Interest

The authors declare that the research was conducted in the absence of any commercial or financial relationships that could be construed as a potential conflict of interest.
